# Advances in upconversion luminescence nanomaterial‐based biosensor for virus diagnosis

**DOI:** 10.1002/EXP.20210216

**Published:** 2022-10-12

**Authors:** Yingjin Ma, Menglin Song, Lihua Li, Xinyue Lao, Man‐Chung Wong, Jianhua Hao

**Affiliations:** ^1^ Department of Applied Physics The Hong Kong Polytechnic University Hong Kong China

**Keywords:** biosensor, luminescent or electric response, pathogen detection, upconversion nanoparticles (UCNPs), virus diagnosis

## Abstract

Various infectious viruses have been posing a major threat to global public health, especially SARS‐CoV‐2, which has already claimed more than six million lives up to now. Tremendous efforts have been made to develop effective techniques for rapid and reliable pathogen detection. The unique characteristics of upconversion nanoparticles (UCNPs) pose numerous advantages when employed in biosensors, and they are a promising candidate for virus detection. Herein, this Review will discuss the recent advancement in the UCNP‐based biosensors for virus and biomarkers detection. We summarize four basic principles that guide the design of UCNP‐based biosensors, which are utilized with luminescent or electric responses as output signals. These strategies under fundamental mechanisms facilitate the enhancement of the sensitivity of UCNP‐based biosensors. Moreover, a detailed discussion and benefits of applying UCNP in various virus bioassays will be presented. We will also address some obstacles in these detection techniques and suggest routes for progress in the field. These progressions will undoubtedly pose UCNP‐based biosensors in a prominent position for providing a convenient, alternative approach to virus detection.

## Graphical abstract

This review discusses the recent advancement in the upconversion nanoparticle (UCNP)‐based biosensors for virus and biomarkers detection. We summarize four basic principles that guide the design of UCNP‐based biosensors, which are utilized with luminescent or electric responses as output signals. Relative strategies with a detailed discussion are presented, and we suggest routes for progress in the field.

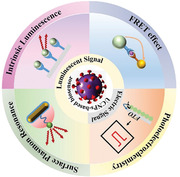

## 1 INTRODUCTION

Diseases caused by infection with viruses, the main cause of mortality worldwide, are emerging and remerging, posing a major threat to people's lives. For example, an acute respiratory illness caused by SARS‐CoV‐2 has traumatized the society for over 2 years. As of March 18, 2022, the World Health Organization (WHO) has reported 464 million confirmed cases and over 6.06 million deaths worldwide. Moreover, the continuous evolution of the SARS‐CoV‐2 variant, from Alpha (B.1.1.7), Beta (B.1.351), Gamma (P.1), Delta (B.1.617.2) to Omicron (B.1.1.529),^[^ ^1^ ^]^ further escalate the difficulties in viral infection diagnosis as it is essential for providing early containment and restorative treatment. Therefore, developing a rapid and reliable pathogen detection methodology is of utmost essential for combating the spread of this infectious disease. More importantly, this high‐speed and definitive detection method provides an opportunity for timely notification to the government and general public to take crucial infectious prevention measures and aid patients in receiving immediate treatment. As is known to all, reverse transcription polymerase chain reaction (RT‐PCR), an amplification technique of specific DNA sequences, is a standard technique for virus diagnostic that has been utilized for decades. RT‐PCR employs fluorescent probes based on the PCR reaction to track the target nucleus acid, allowing for a qualitative analysis of the nasopharyngeal, oropharyngeal, and sputum samples. However, PCR‐based bioassays require tedious sample pretreatment, highly trained operation staff, costly instruments, and prolonged processing time. Furthermore, the possibility of false‐positive results from the PCR inherently restricts the demand for sample collections, storage, and preprocessing.^[^ ^2^, ^3^ ^]^ In terms of immunoassays, enzyme linked immunosorbent assay (ELISA) provides an alternative route for a relatively conventional and effective assay method for detecting antigens, antibodies, and other biomarkers with minimal effort and equipment.^[^ ^4^, ^5^ ^]^ However, time‐consuming processes and cross‐reactivity between antigens need to be further resolved. To tackle this critical issue in reliability while further enhancing the selectivity and sensitivity with a low cost and rapid turnaround time, scientists have developed a variety of innovative biosensors for virus detection. Advances in biosensor‐based virus detection have been made, including magnetic, electronic, electrochemical, microfluidic, and optical‐based biosensors.^[^ ^6^, ^7^, ^8^, ^9^, ^10^, ^11^ ^]^ Especially, optical signal‐based biosensors (e.g., colorimetric, surface‐enhanced Raman scattering, surface plasmon resonance (SPR), and fluorescent biosensors) show good performance in clinic diagnostics thanks to their high sensitivity, stability, specificity, and low background.^[^ ^12^, ^13^, ^14^, ^15^ ^]^

Upconversion nanoparticles (UCNPs) served for competitive optical probes have shown prominent and potential values in biomedical applications, including bioimaging, biosensing, and theranostics done by our group and other researchers.^[^ ^16^, ^17^, ^18^, ^19^, ^20^, ^21^, ^22^, ^23^, ^24^, ^25^, ^26^, ^27^, ^28^, ^29^, ^30^, ^31^ ^]^ The phenomenon of upconversion luminescence (UCL) was originally investigated in the mid‐1960s. Lanthanide–doped upconversion is a nonlinear optical process that converts two or above successive low‐energy photons to a high‐energy photon. UCNPs enjoy outstanding photophysical properties, including high stability, low fluorescence background, low toxicity, long lifetime, sharp emission, and resistance to photobleaching, which is beneficial for improving detection sensitivity.^[^ ^32^ ^]^ The fluorescent lifetime of UCNPs could reach up to tens of milliseconds, which is useful for time‐resolution technology.^[^ ^33^ ^]^ Importantly, the UCL signal could be clearly distinguished from scattering light signal and short‐lived background noise, leading to improving the signal‐to‐noise ratio and greatly enhancing detection capability.^[^ ^34^ ^]^ In addition, UCNPs’ multicolor emission and narrow emission band allow them to perform multiple bioassays while avoiding interference from distinct fluorescence signals.^[^ ^16^, ^35^, ^36^ ^]^ Compared with conventional dyes or quantum dots (QDs) excited by high‐energy UV or short‐wavelength light, UCNPs show more significant superiority in biological field. It is well‐known that biological tissues have an “optical transparency window” in the near‐infrared (NIR) range of 700–1100 nm. A low‐power NIR laser has much less damage to biomolecules (e.g., DNA and cells) under long‐time irradiation than UV or short‐wavelength excitation. Moreover, the penetration depth of tissues under NIR light could reach up to a few centimeters, which is essentially deeper than short‐wavelength excitation. Therefore, utilization of NIR excitation light not only allows for deeper light penetration and minimal photodamage effect, but also offers lower autofluorescence, reduced light scattering, and phototoxicity.^[^ ^37^, ^38^, ^39^ ^]^ Apart from these advantages, UCNPs’ high stability and resistance to photobleaching could provide a more reliable and accurate result under a long‐term detection process. Benefiting from these features, UCNPs as an ideal candidate nanophosphor show great potential in bio‐detection.

**FIGURE 1:**
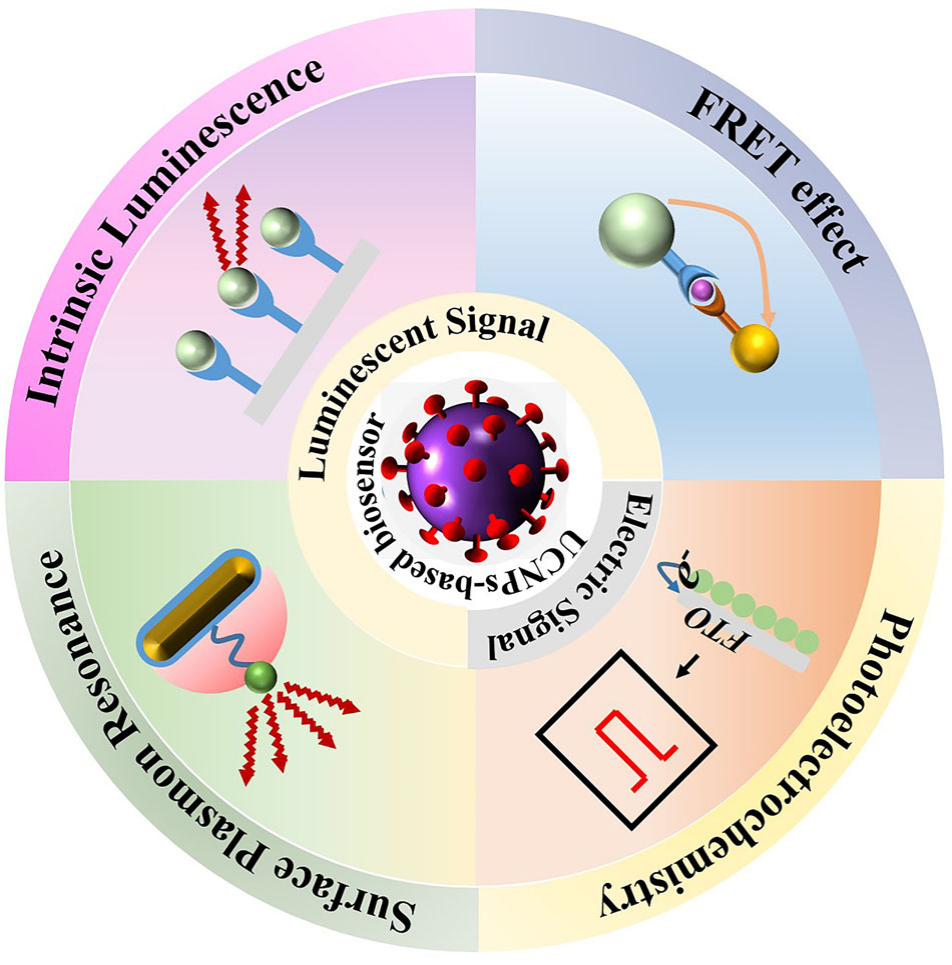
Schematic illustration of various effects used for designing upconversion nanoparticle‐based biosensors.

Nowadays, new strategies such as introducing magnetic and electrochemical techniques are applied to bioassay in combination with UCNPs.^[^ ^40^, ^41^, ^42^ ^]^ In this study, we provide an update on UCNP's recent success in detecting virus or their biomarkers. This review systematically illustrates the design principle and relevant bioassay responses of UCNP‐based biosensors from photoluminescence (PL) to the combination of PL with other effects including Förster resonance energy transfer (FRET), electrochemistry, and SPR. We compare these strategies and present their merits and limitations. We give an overview of the studies on the UC bioassay for virus or biomarkers detection with optical and electrical responses (Figure 1). In addition, UC bioassay in point‐to‐care testing techniques is presented. Finally, some challenges and perspectives for UCNP‐based biosensor applied to virus detection are described.

## 2 FUNDAMENTAL PRINCIPLES AND FIGURES OF METRIC

The design principle of UCNP‐based biosensors mainly depends on the optical properties of UCNPs and the conjugation of UCNPs with other functional particles, which can be mainly classified into four types, namely intrinsic PL, PL with FRET effect, PL with electrochemistry, and PL with SPR effect.

### 2.1 Fundamental principles of biosensing

#### 2.1.1 PL

**FIGURE 2:**
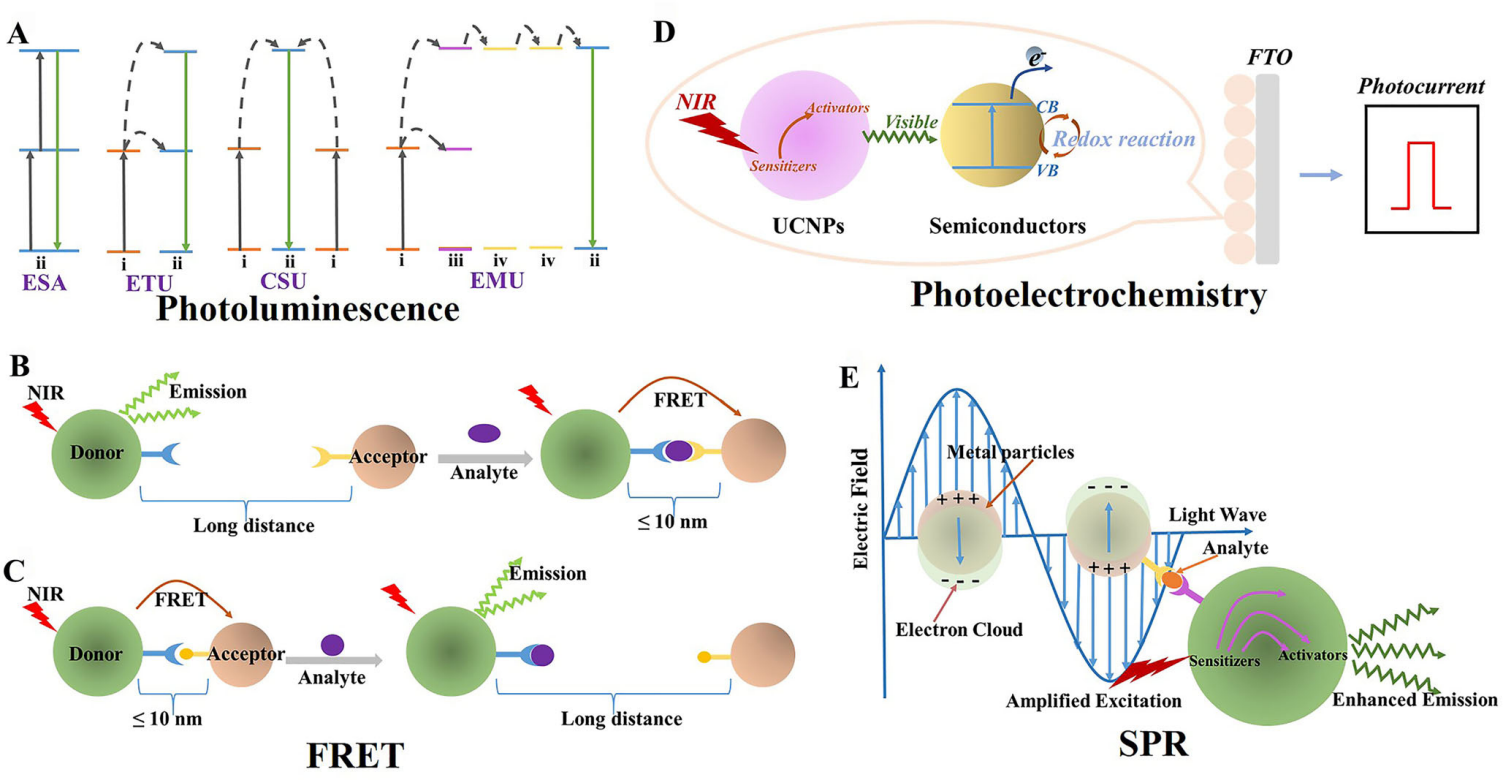
Schematic illustration of four design principles for upconversion nanoparticle (UCNP)‐based biosensor. (A) Four major anti‐Stokes processes of lanthanide–doped NPs; i: sensitizers, ii: activators, iii: accumulators, iv: migrators. (B) Schematic illustration of design principles based on FRET effect. The luminescence is quenched when the donor links closely together with the acceptor upon the addition of analytes. (C) The quenching luminescence is recovered when the donor disconnects with the acceptor upon the addition of analytes. (D) Schematic diagram of the main principle for the PEC biosensors based on UCNPs and semiconductors. (E) Schematic illustration of the design principle based on SPR effect.

Upconversion (UC) PL, one phenomenon from lanthanide ions–doped materials, is fundamental to constructing a platform for sensing biomolecule, which could directly measure the emission intensity to quantify the analytes. In the UC‐based bioassay platform, magnetic separation and point‐of‐care testing are two commonly used techniques to enhance the capability and usefulness of the bio‐detection. The UC PL process mainly arises from the 4f‐4f parity‐forbidden transition of electrons in lanthanide ions, which is relevant to the type, concentration of doping ions, and the structure of host lattice. It usually contains sensitizer (e.g., Yb^3+^) and activators (e.g., Er^3+^, Tm^3+^, Ho^3+^), the sensitizer facilitates the absorption of the photons due to its large absorption cross‐section, which is beneficial for improving UC efficiency. As shown in Figure 2A, the PL process is complicated, depending on four basic mechanisms^[^ ^43^, ^44^ ^]^: i) Excited‐state absorption (ESA), commonly happens in a single activator by successive absorption of multiple infrared photons; ii) Energy transfer upconversion (ETU), contains the energy migration process between two adjacent ions (sensitizer‐activator pair) and is similar to the ESA process; iii) Cooperative upconversion (CUC), involves two sensitizes or more, which could transfer energy to an activator so that the activator is populated to the excited state. It can be observed in the UCNPs with high doping concentration ions; iv) Energy migration upconversion (EMU), usually acts on a core–shell structure nanoparticles (NPs), which includes accumulators and migrators except for the sensitizers and activators. The sensitizers could transfer the energy to accumulators, and then the stored energy in the accumulators could be taken by the migrators until it is trapped by activators, leading to the irradiation relaxation of activators. Designing the appropriate type and concentration of doping ions is key for enhancing the UC luminescent efficiency, further increasing the sensitivity in bioassay. In UCNP‐based bioassay, functionalized‐UCNPs served for fluorescent probes can capture targets due to their specific recognition, such as antigen‐antibody reaction and base complementary principle. When UCNP probes are conjugated with targets, the reacted UCNPs are separated from the unreacted by various methods. Consequently, the UCL characteristic (e.g., intensity) as the readout signal can be utilized to analyze the detection targets qualitatively and quantitatively. For example, magnetic materials as probes conjugate with UCNP probes upon the presence of targets, and the reacted UCNPs could separate with the unreacted ones under an extra magnetic field. Alternatively, in lateral flow assay strips or microarrays, the UCNP probes can be captured in a specific position and excess UCNPs will be removed by the flow rate or washed. As a result, the UCL intensity of reacted UCNPs can be directly detected as the output signal to demonstrate the concentration of the targets. Therefore, UCL intensity is a direct signal for target detection in UCNP‐based biosensors, which can be an effective method to specially quantify the targets.

#### 2.1.2 FRET

The principle of most UCNP‐based bioassays relies on FRET, which reports multiple signals such as optical and electric signals. FRET, also known as fluorescence resonance energy transfer or luminescence resonance energy transfer (LRET), was initially proposed in the late 1940s by Förster and provides the advantages of rapid response and multiplexing capability with small quantity of samples.^[^ ^45^ ^]^ The FRET process is a non‐radiative energy transfer involving two long‐range dipole‐dipole coupling in which the excited state of donors transfers its energy to the adjacent ground state of acceptors. This process quenches the fluorescent signal from the donors and the acceptors will be excited if the acceptor is emissive.^[^ ^29^, ^45^, ^46^ ^]^ Basically, the FRET efficiency depends on several elements as described in the Förster equation:

(1) $$E_{\mathrm{FRET}}=\frac{{R_{0}}^{6}}{{R_{0}}^{6}+r^{6}}\;$$

(2) $${R_{0}}^{6}=\frac{9\left(\ln10\right)\kappa^{2}\Phi_{D}T}{128\pi^{5}n^{4}N_{A}}\;$$

where *R*_0_ is the Förster distance, *r* is the donor‐acceptor distance, κ^2^ is the orientation factor, Φ_D_ is the quantum yield of donors, T is the spectral overlap between donor and acceptor. Hence, there are two key factors in the FRET process, namely i) overlap between the emission spectrum of donors and absorption spectrum of acceptors, the energy can be transferred due to the matching energy level, and ii) The distance between donors and acceptors, FRET is a distance‐dependent process and the maximum distance between donor‐acceptor pairs is up to 10 nm.^[^ ^47^, ^48^ ^]^ Moreover, the length scale of many biomolecules and most biological processes (e.g., protein‐protein interaction) match the FRET requirements, making FRET an effective tool for probing biomolecules.^[^ ^49^, ^50^ ^]^ In the FRET process, it is essential to rationally design and choose the types of donor and acceptor. Interestingly, UCNPs with multiple emission bands, sharp emission peaks, long lifetime, and absence of autofluorescence, usually act as donors, which shows a promise in FRET‐based biosensors. Whereas, there are various choices for acceptors, such as Au NPs,^[^ ^51^, ^52^ ^]^ semiconductors QDs (QDs),^[^ ^53^, ^54^ ^]^ organic dyes,^[^ ^55^, ^56^ ^]^ graphene oxidation (GO),^[^ ^57^, ^58^ ^]^ and graphene QDs (GQDs),^[^ ^45^, ^59^ ^]^ whose absorption spectra are requested to overlap well with the emission spectrum of donors, which could be used in the UCNP‐based biosensor on a basis of the FRET process. The relative properties of those acceptors are summarized in Table 1. Generally, the design diagram of UCNP‐based biosensors for biomolecules (i.e., nucleic acid, antigen, and antibody) detection may be divided into two categories, as shown in Figure 2B,C: i) Analytes as a linker conjugate the functionalized donors and acceptors in a close distance, causing the luminescent quenching of donors and such a quenching effect rises with the increase of analyte concentration; ii) the presence of analytes will destroy the connection between donors and acceptors and cause one of them to develop a more stable structure, thus inducing the recovery of luminescence.

**TABLE 1:** Comparison of commonly used donor‐acceptor pair in upconversion nanoparticles‐based biosensors

Nanoprobes	Donor‐acceptor pair	Advantages	Disadvantages	Ref.
Metal Au	Acceptor	High luminescence quenching capability Easy surface modification	Aggregation	^[^ ^51^, ^52^ ^]^
Semiconductor QDs	Acceptor	Broad absorption spectra High quantum yield High molar extinction coefficients Photostability	Autofluorescent Spectral cross‐talk Toxic	^[^ ^53^, ^54^ ^]^
Organic dyes	Donor or acceptor	Small molecular volume High bioselectivity High fluorescence efficiency Good biocompatibility	Photobleaching Spectral cross‐talk High background fluorescence	^[^ ^55^, ^56^ ^]^
GO	Acceptor	Broad absorption band Low cost Good water solubility	Poor control over the dimension and surface chemistry	^[^ ^57^, ^58^ ^]^
GQDs	Donor	Finite band gaps Good solubility Ease of synthesis and modification	Low luminescence quantum yield	^[^ ^45^, ^59^ ^]^

#### 2.1.3 Photoelectrochemistry

Photoelectrochemistry contains a light‐to‐electric transition process and an interconversion process of electric energy to chemical energy, which is a newly emerged analytical technique in biosensing. UCNPs in photoelectrochemical (PEC) sensing commonly combines with semiconductors as a new type of photoactive material, which overcomes the broadband barrier of semiconductors. As shown in Figure 2D, UCNPs absorb photons under the excitation source (NIR) to generate emission in the ultraviolet‐visible range. Subsequently, semiconductors with a well‐matched energy level could be excited by the photons generated from UCNPs, leading to electron–hole pair generation.^[^ ^60^, ^61^ ^]^ The UCNPs combine with semiconductors in various strategies to form an integrated photoactive material, which effectively improves the light‐to‐electric transmission efficiency. In PEC biosensing, the photoactive materials are on the surface of the working electrode, which could form a photocurrent signal by transferring the photo‐generated electrons after absorbing photons and cause the redox reaction from the biological recognition reaction in the solid‐liquid interface due to the efficient energy conversion. When the analytes (antigen/ antibody and nucleic acid) are recognized, it has a direct or indirect influence on photoactive materials or electrolytes, which causes the variation of photocurrent signal with the concentration of the analytes.

#### 2.1.4 SPR

SPR is a collective oscillation phenomenon in which the vibration frequency of free electrons on the surface layer of metal is coupled with the incident photons, as shown in Figure 2E. It may generate a strong electromagnetic field around the surface of the metal (e.g., Au, Ag), which amplifies the excitation light. Coupling plasmonic metal materials with UC materials could largely increase the UC emission in the following three ways.^[^ ^62^, ^63^, ^64^ ^]^ i) Enhancing the absorption of sensitizers from electrical field coupling, it is noted that photo flux of excitation has a square relationship with the SPR induced electric field so that it can improve the sensitizer's photons harvest efficiency when coupling transition dipole of UCNPs with plasmon, resulting in the luminescent enhancement. ii) Improving the radiative rate of activators, the quantum yield of UCNPs is relative to the decay rate by the equation:

(3) $$\varphi \;=\frac{k_{r}+k_{m}}{k_{m}+k_{m}+k_{\mathrm{nr}}}\;$$

where *k*_r_ is the radiative decay rate. Therefore, the UC emission is improved by the radiative decay rate. iii) Increasing energy transfer efficiency from sensitizers to activators, because the surface plasmon‐enhanced luminescence is proportional to the energy transfer. Importantly, the SPR is dependent on the shape, and size of noble metal, and furthermore the distance between UCNPs and plasmonic metal influences the UC emission intensity. Therefore, SPR provides a novel platform for bio‐detection by monitoring the enhanced signal of UC emission, in which UCNPs and metal plasmon act probes and bind together with the presence of targets, and the UC emission enhances with an increase in target concentration.

### 2.2 Figures of merit

The figures of merit are routinely used to assess the sensor performance, which employs the quantifiable terms to show the quality of the sensing process and hence guarantee the quality of outputs. It is beneficial for developing the performance of biosensors and be further utilized in clinical applications. Typically, key figures of merits for evaluating biosensors include sensitivity, limit of detection (LOD), specificity, selectivity, repeatability, and reproducibility.^[^ ^65^, ^66^ ^]^ Sensitivity, measured as the slope of the analytical calibration curve, refers to the response ability to detect analytes. If a biosensor features high sensitivity, it indicates that a tiny change in the concentration of analytes will result in a significant variation in response signals. Selectivity is the specific recognition ability of sensors that distinguish the response of analytes from that of others. It is defined as the slope of a target's calibration curve divided by the slope of other interference analyte's calibration curve. LOD is the lowest concentration of analytes that can be detected by the signal response with reasonable certainty. The LOD value is calculated based on the equation:

(4) LOD=3Q/S

where *Q* and *S* are the standard deviation of the blank sample and the slope of the calibration curve, respectively. Repeatability and reproducibility are the closeness of the agreement between the results of successive measurements under identical or different conditions, which are influenced by relative factors such as instruments, operators, and laboratories.

## 3 UC BIOASSAY FOR THE DETECTION OF VIRUS

Nucleic acid and structural protein (e.g., antigen) are two important specific characteristics of viruses' genetic and structural information. In addition, antibody, a protective protein produced in the body after antigen stimulation, is also a specific biomolecule in virus detection. Therefore, nucleic acid, antigen, and antibody are three commonly used recognition targets in virus detection in which they can be detected by complementary base pairing principle and antigen‐antibody reaction, respectively. Table 2 summarizes previously reported UCNP‐based biosensors which are involved with their size, bioconjugation, target, LOD, linear range, and effect on DNA/RNA or antigen/antibody detection.

**TABLE 2:** Summary of upconversion nanoparticle‐based biosensor

Type of UCNPs	Size (nm)	Bioconjugation	Target	LOD	Linear range	Effect	Ref.
NaGdF_4_:Yb,Er@NaYF_4_	34	Oligonucleotide	HIV DNAs	15 pM	0.02–30 nM	PL	^[^ ^68^ ^]^
NaY_0.78_F_4_:Yb_0.2_,Er_0.02_	35	Oligonucleotide	miR‐21‐5p	50 fM	—	PL	^[^ ^69^ ^]^
NaYF_4_:Er/Yb@NaYF_4_;NaYF_4_:Tm/Yb@NaYF_4_	50	Antibody	PSA/EphA2	60–400 pg/ml	60–400 pg/ml	PL	^[^ ^70^ ^]^
NaYF_4_:Yb,Er;NaYF_4_: Yb,Tm	54	Aptamers	Salmonella	10 ng/ml	0.01–50 μg/ml	PL	^[^ ^74^ ^]^
NaGdF_4_:Yb,Er@NaGdF_4_	22.4–30.5	Antibody	Cephalexin	10 ng/ml	0.5–100 g/ml	PL	^[^ ^75^ ^]^
—	—	Antibody	Hepatitis B virus	0.1 IU/Ml	—	PL	^[^ ^76^ ^]^
—	68	Antigen	HIV‐1/2 antibodies	—	—	PL	^[^ ^77^ ^]^
NaYF_4_:Yb,Tm@NaYF_4_:Ca	45	Antibody	AIV	10^3.5^EID_50_/ml	102.5–105 EID_50_/ml	PL	^[^ ^78^ ^]^
NaYF_4_:Yb,Er@NaYF_4_@mSiO_2_	75.2	Antibody	SARS‐CoV‐2	1.6–2.2 ng/ml	2–200 ng/ml	PL	^[^ ^79^ ^]^
NaGdF_4_@NaYF_4_:Yb,Er@NaYF_4_	17.7	Aptamer	Influenza A viruses	60.9 pg/ml	0.1−15 ng/ml	FRET	^[^ ^57^ ^]^
NaGdF_4_:Yb,Er	21	Oligonucleotide	Alpha‐fetoprotein	0.059 aM	1–100 aM	FRET	^[^ ^71^ ^]^
—	—	Oligonucleotide	PSA	0.032 × 10^−18^ m	(0.04–1) × 10^−18^ m	FRET	^[^ ^72^ ^]^
NaGdF_4_:Yb/Er@NaGdF_4_:Yb/Nd	—	Oligonucleotide	Influenza H7 subtype	134 × 10^−12^ m	100 × 10^−12^ ‐ 1 × 10^−9^ m	FRET	^[^ ^94^ ^]^
BaGdF_5_:Yb/Er	14	Oligonucleotide	AIV H7 subtype	7.0 pM	10 pM–10 nM	FRET	^[^ ^89^ ^]^
NaYF_4_:Yb,Er	48	Oligonucleotide	Hepatitis B virus	250 pM	0–50 nM	FRET	^[^ ^90^ ^]^
NaYF_4_:Yb,Er	60	Peptide	HIV Antibody	2 nM	5–150 nM	FRET	^[^ ^92^ ^]^
β‐NaYF_4_: Er^3+^ Yb^3+^/β‐NaYF_4_	25.2	Streptavidin	SMN1 DNA	34 fmol	—	FRET	^[^ ^91^ ^]^
NaYF_5_:Yb,Er	30	Oligonucleotide	HIV DNAs	3 nM	0–80 nM	FRET	^[^ ^93^ ^]^
NaYF_4_:Yb,Er@SiO_2_	32	Oligonucleotide	DENV‐2‐vsrna5	10 fM	—	FRET	^[^ ^95^ ^]^
BaGdF_5_:Yb/Er	14	Oligonucleotide	Ebola virus	300 fM	50–700 fM	FRET	^[^ ^97^ ^]^
NaYF_4_:Yb,Tm@TiO_2_@CdS	—	—	miRNA‐133a	36.12 aM	0.1 fM–1 nM	PEC	^[^ ^98^ ^]^
NaYF_4_:Yb,Er UCNPs@CdTe	—	Aptamers	CEA	4.8 pg/ml	10 pg/ml–5 ng/ml	PEC	^[^ ^99^ ^]^
NaYF_4_:Yb,Tm@TiO_2_	—	—	CEA	3.6 pg/ml	10 pg/ml–40 ng/ml	PEC	^[^ ^100^ ^]^
NaYF_4_:Yb^3+^,Er^3+^@Au@CdS	361	Antibody	Alpha‐fetoprotein	5.3 pg/ml	0.01−40 ng/ml	PEC	^[^ ^101^ ^]^
NaGdF_4_:Yb,Er	20	Oligonucleotide	DENV‐2‐vsRNA5	20.3 aM	3.3 × 10^−8^–3.3 × 10^−5^ nM	SPR	^[^ ^102^ ^]^
NaYF_4_@NaYF_4_:Yb, Er@NaYF_4_	25	Oligonucleotide	MicroRNA	0.036 fM	10^−16^–10^−10^ m	SPR	^103^

*Note*: PL in this table is the abbreviation of photoluminescence; PL in refs. [68, 69] based on magnetic separation technique; PL in the refs. [70, 74, 75, 76, 77, 78, 79] based on LFIA.

### 3.1 Bioassay through UC luminescent response

#### 3.1.1 Magnetic separation

**FIGURE 3:**
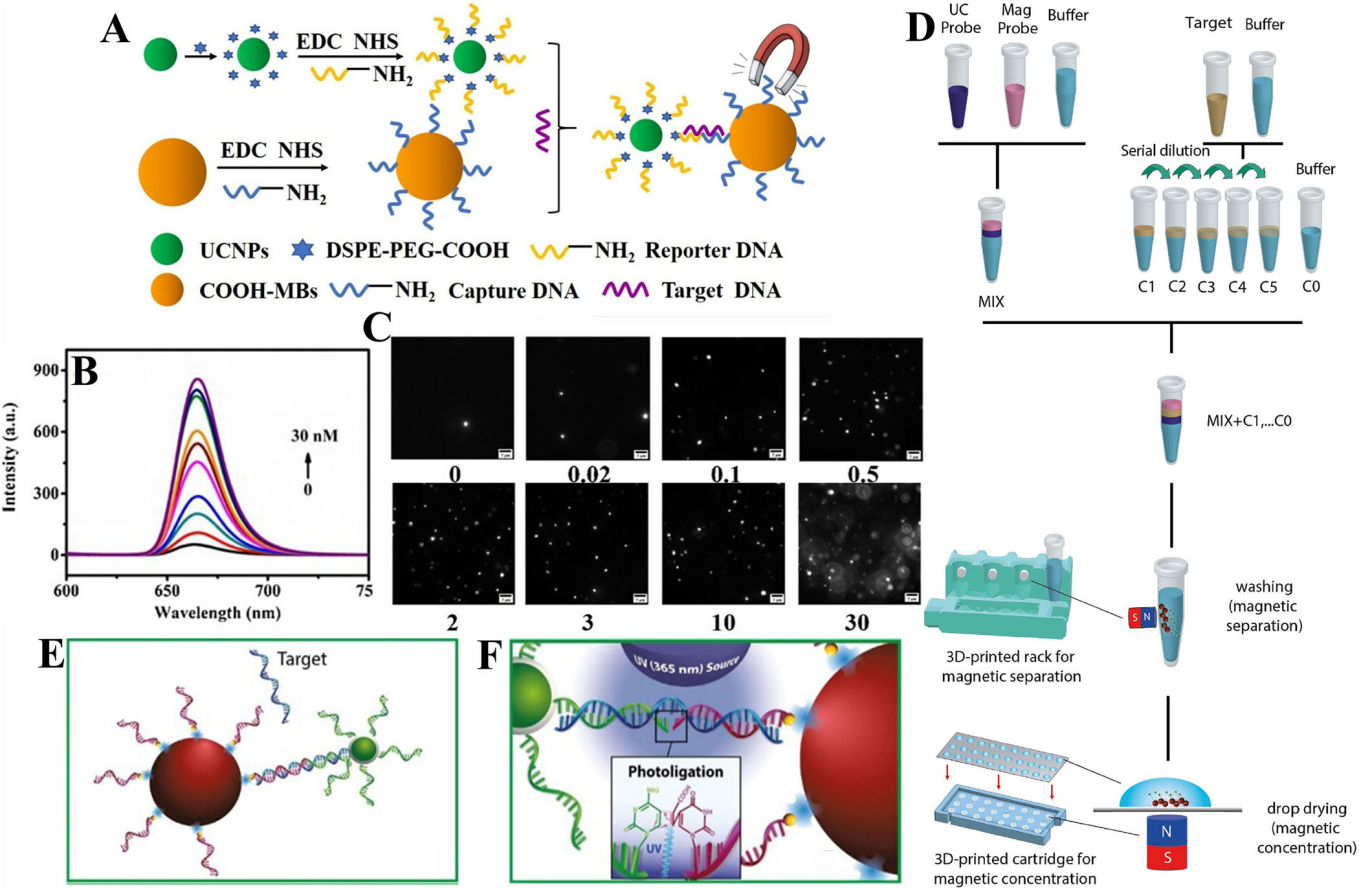
Schematic illustration of upconversion nanoparticles (UCNPs)‐based biosensors with magnetic separation mechanism. (A) Schematic illustration of the sandwich structure for HIV DNA detection. (B,C) Luminescent spectra and quantitative image analysis of UCNPs probes with different HIV‐1 concentrations. Reproduced with permission.^[^ ^68^ ^]^ Copyright 2021, Elsevier. (D) Procedure for miR‐21‐5p detection on a 3D printed assay setup. (E,F) The target is captured with UCNPs probe and magnetic probe and its relative photoligation reaction between these ssDNA strands under UV irradiation, respectively. Reproduced with permission.^[^ ^69^ ^]^ Copyright 2021, Elsevier.

Magnetic separation is a widely used technique in biosensors because it is capable of effectively isolating and separating biomolecules without the need for complicate and tedious extraction processes such as repeatable filters and centrifuges.^[^ ^67^ ^]^ UCNPs with zero background signal and high‐energy fluorescent emission are useful for signal probes in biosensors. The UCNPs could conjugate with magnetic materials with the existence of the target and thus be separated by the external magnetic field, resulting in a change of luminescent intensity in the detection solution. Therefore, the connection of UCNPs and the magnetic materials provides a target detection platform enjoying the merits of simple operation, time‐saving, and high sensitivity, leading to its usefulness in DNA/RNA, antigen, and enzyme bio‐detection. Based on the excellent separation ability and significant change of luminescent intensity, Tang et al. developed a sandwich strategy based on NaGdF_4_: Yb, Er@NaYF_4_ UCNPs, and magnetic beads (MBs) for HIV DNAs detection (Figure 3A–C).^[^ ^68^ ^]^ The target could be recognized and separated by forming a UCNPs‐HIV DNA‐MBs structure according to the complementary base pairing principle. And under magnetic separation, UCL can be measured in the separated material. The quantitative image analysis was introduced in this study, and an increase of photons was observed after adding targets, which showed a lower LOD and a wider linear range than that of the UCL signal. Benefiting from the high sensitivity and rapidity, this strategy is capable of being extended to the detection of other viruses.

In order to improve the detection sensitivity, Diego et al. fabricated a 3D‐printed portable assay device based on NaY_0.78_F_4_: Yb_0.2_, Er_0.02_ NPs with γ‐Fe_2_O_3_ magnetic microparticles for ultrasensitive detection of miR‐21‐5p DNA‐analogue (Figure 3D–F).^[^ ^69^ ^]^ With the presence of targets, it not only removes the unreacted UCNPs from the solution after three washes by magnetic separation, but also concentrates the reacted complex into a small spot on glass, proving the advantages of detecting UCL intensity with a small amount of targets. In this work, a photoligation reaction was introduced to form a covalent link between different ssDNA, which prevents the UCNPs from dissociating from magnetic materials after several times of washes and thereby improves the efficiency of target recognition. Based on the concentration and photoligation process, the fabricated UCNP‐based portable biosensor presented rapid detection and high sensitivity with a low LOD of 0.05 pM, which improved more than 10‐folds better than those without photoligation step. Therefore, the combination of magnetic NPs and UCNP‐based sensors is promising in viruses and biomolecules assays, which might be a step forward in developing a point‐of‐care diagnostic platform.

#### 3.1.2 Point‐of‐care testing

**FIGURE 4:**
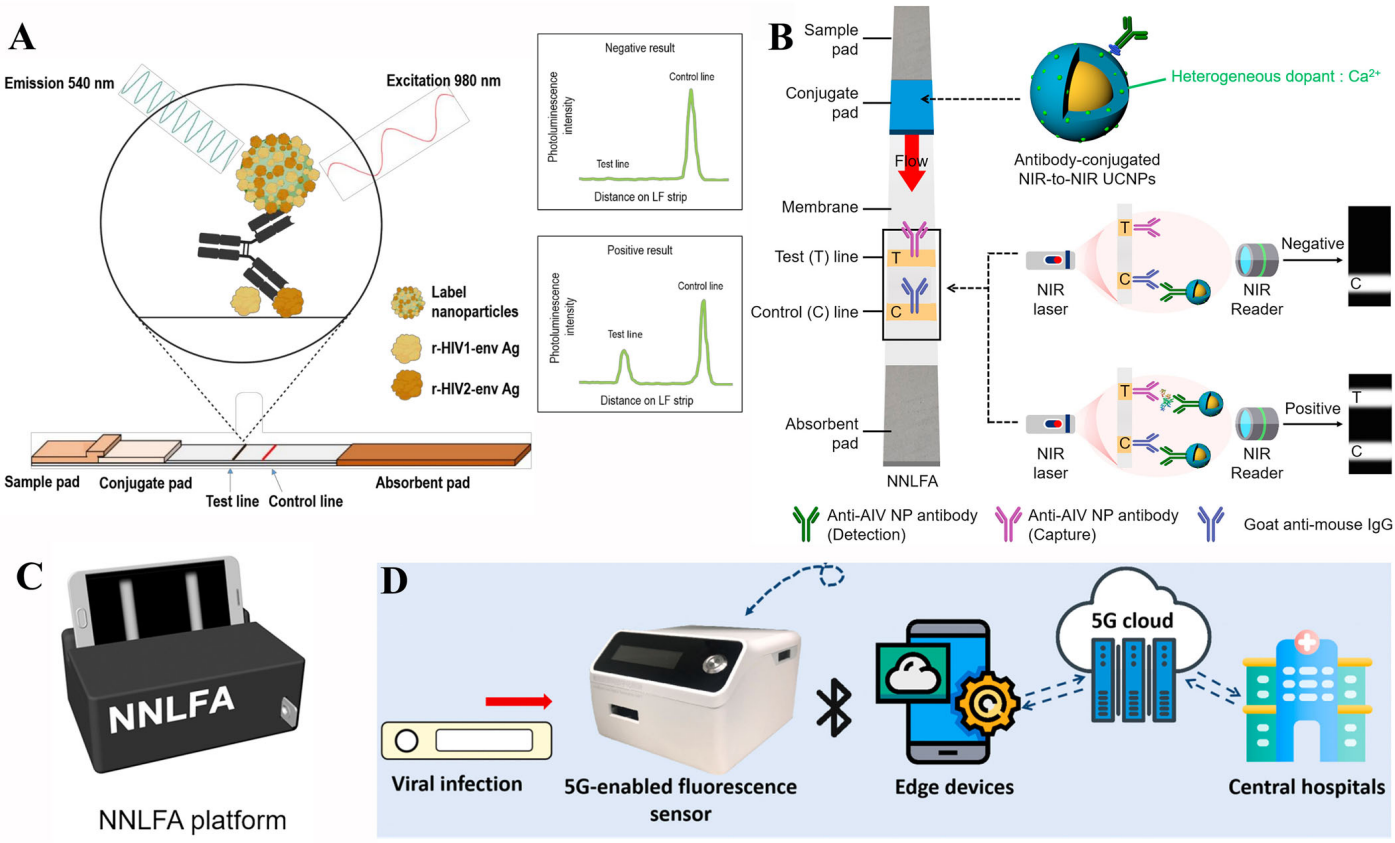
Schematic illustration of upconversion nanoparticle (UCNP)‐based LFIAs for various virus detection. (A) The double‐antigen bridge design principle of UCNP‐based LFIAs for anti‐HIV‐1/2 antibodies detection. Reproduced with permission.^[^ ^77^ ^]^ Copyright 2021, Multidisciplinary Digital Publishing Institute. (B) The strip design and measurement principle of near‐infrared‐to‐near‐infrared UCNP‐based LFIAs for AIV detection. (C) A portable UCNP‐based detection platform is developed for readout. Reproduced with permission.^[^ ^78^ ^]^ Copyright 2018, Elsevier. (D) The working process of the 5G‐enabled fluorescent sensors. Reproduced with permission.^[^ ^79^ ^]^ Copyright 2021, Elsevier.

Point‐of‐care testing (POCT), also known as near‐patient testing, is an on‐site clinic medical test that can be performed without the need to send a sample to a laboratory in a short period of time. It can not only assist caregivers with immediate diagnosis and clinical intervention but also be applied in high‐throughput detection and in some developing regions without expensive instruments. Lateral flow immunoassays (LFIAs), also known as immune chromatographic strip test (ICST), is a paper‐based bioanalytical platform for one‐site target detection. It has been widely used in various viruses and diseases detection due to its excellent characteristics of affordability, user‐friendly, equipment‐free, sensitivity, and short turnaround time.^[^ ^73^ ^]^ In comparison with traditional visual labels (e.g., colloidal gold, carbon NPs, and dyes), UCNPs show the potential of an alternative fluorescent label serving for LFIAs in terms of sensitivity improvement, which allows for direct monitoring of the luminescent intensity by the target concentration during the biological recognition process.^[^ ^74^, ^75^, ^76^ ^]^ LFIAs based on UCNPs could attain high visibility, produce quantitative testing and avoid misinterpreting the result caused by inadequate signal generation of the traditional labels, resulting in the potential of being applied in high‐throughput testing. Benefiting from the convenience of LFIAs and the high luminescent intensity of UCNPs, Martiskainen et al. presented a UCNP‐LFIAs based on a double‐antigen bridge structure for anti‐HIV‐1/2 antibodies detection (Figure 4A).^[^ ^77^ ^]^ A portable reader setup was used in this experiment for automating result interpretation, readily archiving and transferring data from decentralized locations, which is much more convenient. In their strategy, the target samples are obtained from plasma and serum of patients to evaluate the UCNPs‐LFIAs performance, which presents reference value in clinic diagnosis and could extend to other viruses’ detection. The targets in this biosensor could be detected within 30 min and it has high sensitivity and specificity in HIV‐1 detection.

To address the problem of low sensitivity in opaque solutions, Kim et al. developed a NIR‐to‐NIR based LFIAs based on NaYF_4_:Yb,Tm@NaYF_4_:Ca NPs for avian influenza virus detection (Figure 4B).^[^ ^78^ ^]^ The doping of Ca^2+^ ions in small‐size UCNPs greatly increased the luminescent intensity of UCNPs. In this strategy, the emission in the NIR region avoids the visible signal interference from opaque stool samples, UCNPs, and some assay membrane. Therefore, the sensitivity and accuracy of the assay can be improved. Making use of the high NIR PL and assistance of a simple readout device, the UCNP‐based LFIAs showed a low LOD of 10^2^ EID_50_/ml for H5N2 and 10^3.5^ EID_50_/ml for H5N6, a tenfold increase in sensitivity over commercial LFIAs (Figure 4C). Guo et al. developed a rapid UCNP‐LFIAs with a 5G‐enabled readout device for the detection of spike protein and nucleocapsid protein of SARS‐COV‐2 (Figure 4D).^[^ ^79^ ^]^ An innovation of their approach is the introduction of the internet of medical things, providing a way to output the test results connected with edge device and fog layer of the internet within 10 min. The hospital might gather and evaluate these medical records, which would be valuable for illness prevention. But the LOD of these LFIAs is not sensitive enough. Therefore, UCNP‐LFIAs is a rapid, convenient and high‐throughput technique, which has certain practical application values, especially in remote areas without advanced medical testing equipment and enough medical resources. However, the sensitivity of LIFAs is relatively much lower than the PCR technique and may cause false positive results. And the opaque plasma and serum samples have an impact on the measured luminescent intensity, resulting in influencing the achieved sensitivity. Moreover, UCNPs‐LFIAs need a readout device equipped with a NIR excitation source, which may increase the cost.

#### 3.1.3 Microarray

**FIGURE 5:**
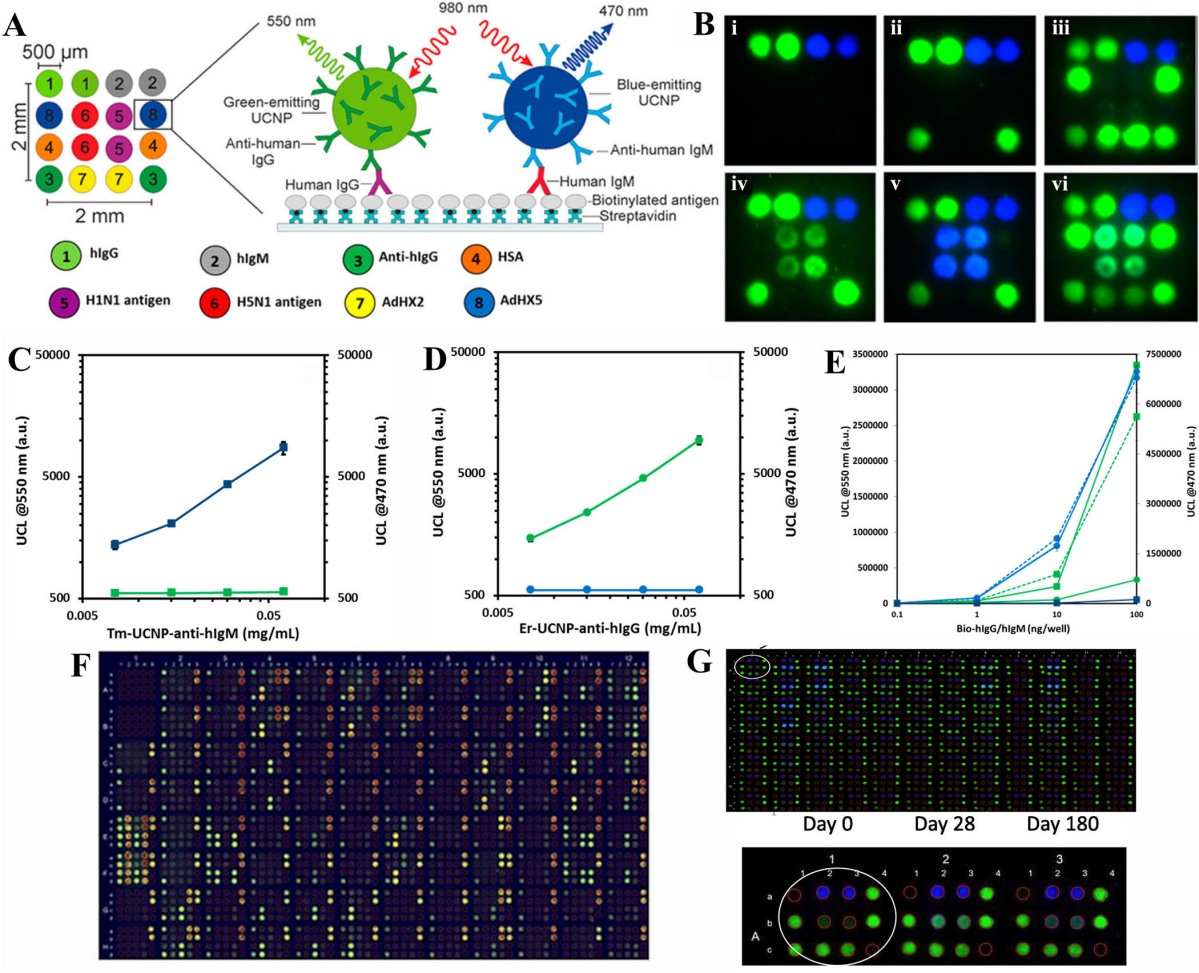
Schematic illustration of upconversion nanoparticle (UCNP)‐based microarray. (A) The design principle of array‐in‐well microarray based on UCNPs and various antigens. (B) The upconversion luminescence (UCL) image of array areas with i: no sample, ii: negative sample, iii: anti‐adenovirus positive sample, iv: anti‐influenza IgG positive sample, v: anti‐influenza IgM positive sample, and vi: IgG positive sample for both adenovirus and influenza. (C,D) UCL spectrum with different Tm‐UCNP‐anti‐hIgM and Er‐UCNP‐anti‐hIgG, respectively. (E) Cross‐reactivity of UCNP antibody conjugates. Green (550 nm, Er‐UCNP); blue (470 nm, Tm‐UCNP). Reproduced with permission.^[^ ^82^ ^]^ Copyright 2016, American Chemical Society. (F) The overall visualization of fluorescent image in a 96‐well plate. Reproduced with permission.^[^ ^85^ ^]^ Copyright 2019, American Society For Microbiology. (G The overall fluorescent image in one 96‐well plate and serum sample collected from same individual at days 0, 28, and 180. Reproduced with permission.^[^ ^86^ ^]^ Copyright 2020, Elsevier.

Microarrays are a dual‐dimensional array of biological probes in a solid substrate by specific recognition (e.g., base complementary principle, antigen‐antibody), which could use a slight trace of samples for the detection with high efficiency, fast speed, and low cost. It is an emergency high‐throughput bioanalytical method with several biomarkers in a single array that can prevent infectious diseases, which is increasingly adopted in clinical diagnostics.^[^ ^80^, ^81^, ^82^ ^]^ Since the 1990s, DNA microarrays have been proven to be a powerful gene expression analysis method. Because of the limited information apart from the gene itself, the protein microarray was developed to investigate the multiplex functions of organisms.^[^ ^83^ ^]^ The multi‐analyte array contains the planar array format where the analyte is recognized by its location and the microbead‐based array format where the analyte is identified by the color of microbeads. Although the latter is more extensively utilized due to its high surface‐to‐volume ratio, it necessitates a more sophisticated apparatus than the former.^[^ ^84^ ^]^ Organic dyes and QDs are the commonly used fluorescent signals in this assay, whereas dyes suffer from the problem of intense background signal, photobleaching, poor photostability, and multiple excitation sources needed in the test. Furthermore, QDs are readily influenced by the autofluorescence of biological matrices. In contrast to conventional fluorescent signals, UCNPs have high photostability, multiple emission bands with different doping ions, and sharp emission peaks, which avoids signal interference. Benefiting from the high‐energy luminescence of UCNPs and the high‐throughput ability of microarray, Kale and co‐workers developed a dual‐mode array‐in‐well assay based on NaYF_4_:Yb^3+^, Er^3+^ and NaYF_4_:Yb^3+^, Tm^3+^ for detecting serum IgG and IgM antibodies against influenza A and human adenoviruses (Figure 5A).^[^ ^82^ ^]^ Several types of viral antigens and human IgG, IgM in an array format were printed in the microtiter wells after the modification of streptavidin, and thus the type of antibodies against different viruses was confirmed by the position and wavelength of UCNPs’ luminescent signal (Figure 5B). The result of spectral cross‐talk testing and the cross‐reactivity testing (Figure 5C–E) revealed that anti‐hIgM/anti‐hIgG modified Tm/Er‐UCNP had a high specific binding to hIgM/hIgG. It indicated that this technique enjoyed high specificity without the interference of spectral cross‐talk. In recent years, Kazakova et al. developed a similar array‐in‐well microarray in a 4 × 5 format based on UCNPs for antibody detection against various respiratory syncytial viruses in young children (Figure 5F).^[^ ^85^ ^]^ The overall visualization of the microarray layout simultaneously showed the signal of tens of samples. The fluorescent color on the signal spot was related to the signal counts, indicating that microarray is a reliable method as a high‐throughput technology. To investigate the effect of the vaccine, Kazakova and co‐workers subsequently proposed a dual‐mode multiplex microarray for influenza‐specific IgG and IgM antibodies detection of adults with or without Pandemic vaccination (Figure 5G).^[^ ^86^ ^]^ The fluorescent array‐in‐well image and specific signal counts showed the different antibodies’ responses after vaccination within half a year. These works indicated that multiple microarrays are a reliable tool to investigate large‐scale seroprevalence, providing crucial information to vaccine researchers. Therefore, the utilization of microarray for antibodies detection is an effective method of data analysis for infectious diseases, and it can estimate the immune status of antibodies against vaccination or virus and the prevalence of viral diseases, which is a useful approach to control the spread of viruses and great value in clinic diagnosis. However, microarray has some limitations that influence the accuracy of analytical results. For instance, the distribution of UC luminescent intensity on the edge and inner spots is different due to the surface intension and high evaporation rate on the edge.

### 3.2 FRET‐based bioassay

It is accepted that FRET is a rapid and sensitive bioanalysis technique with great potential for various biomolecules and virus detection. Conventional downconversion materials, such as QDs and organic dyes, are facing the problem of strong background fluorescent signal in the assay and a low signal‐to‐noise ratio, which limits the sensitivity of the assay. As compared to these shortcomings of downconversion materials, UCNPs as signal probes show a rapid response, sharp emission, photostable, good biocompatibility, and high signal‐to‐noise ratio which facilitates improving the sensitivity. More importantly, NIR light source employed in pumping UCNPs enjoys low photodamage to the virus oligonucleotide hybridization with probes, compared with traditional downconversion counterparts. Based on these characteristics intrinsically possessed in UCNPs, FRET‐based biosensors incorporated with UCNPs are potentially used in virus detection considering their outstanding sensitivity, rapid response, and low cost. Normally, the FRET‐based assay can be divided into two types, including homogenous and heterogeneous assays, which depend on the separation of unbound labeled analyte prior to measuring bound signal. Heterogeneous assays require multiple washing procedures to remove unbound components caused by the antibody or analyte being immobilized on a solid phase. Comparatively speaking, no washing or separation processes are necessary for homogeneous assays, since the signal is generated only when the binding reaction occurs.^[^ ^87^ ^]^

#### 3.2.1 Homogeneous assay

**FIGURE 6:**
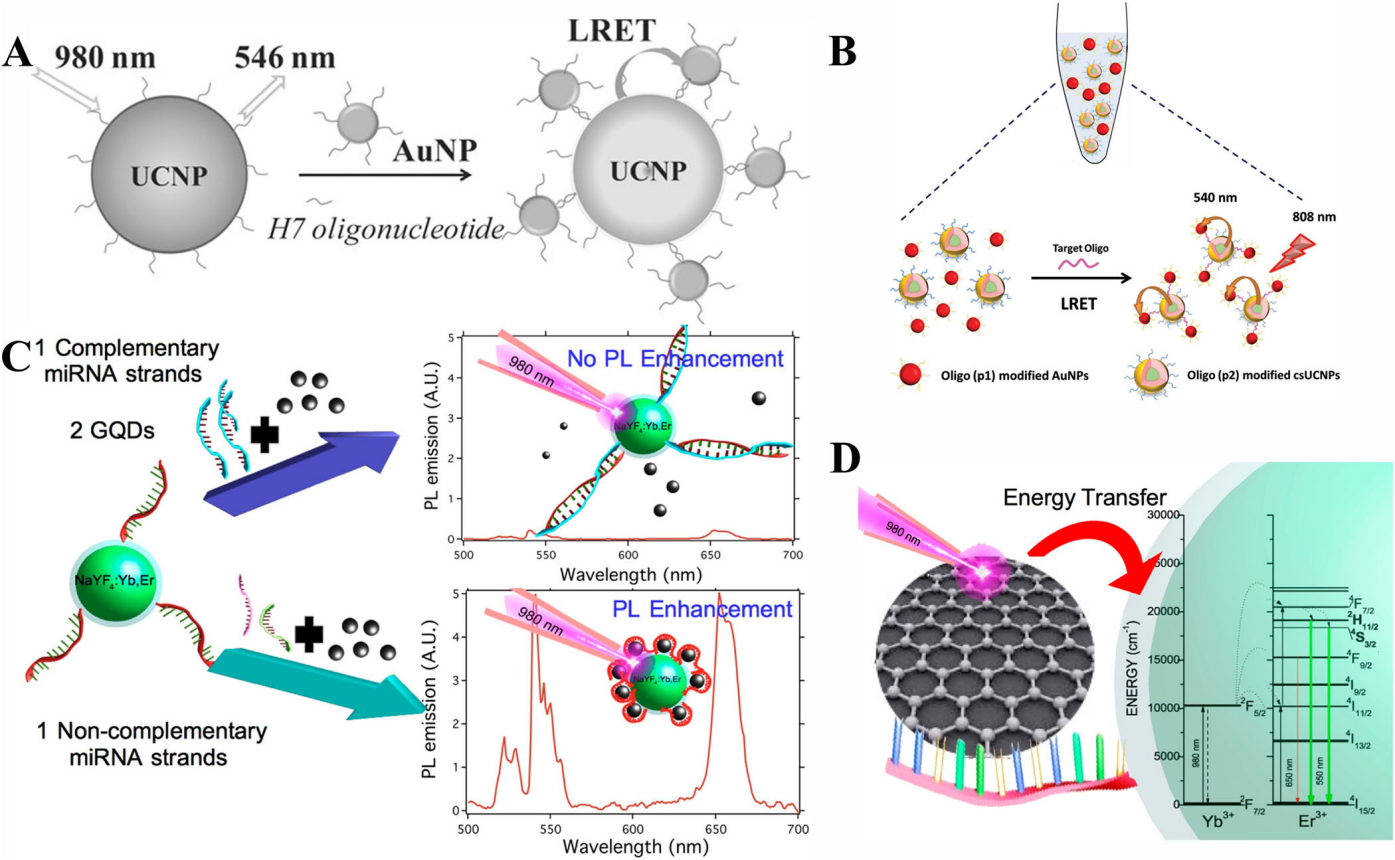
Schematic illustration of homogeneous upconversion nanoparticle (UCNP)‐based biosensors with FRET effect. (A) The design principle based on UCNPs and Au NPs for avian influenza virus H7 subtype detection. Reproduced with permission.^[^ ^89^ ^]^ Copyright 2014, Wiley Online Library. (B) Homogeneous sandwich detection scheme based on Au NPs and UCNPs. Reproduced with permission.^[^ ^94^ ^]^ Copyright 2019, Wiley Online Library. (C) Design principle of the biosensor with or without complementary miRNA oligonucleotides, the black balls in this figure represented GQDs. (D) The energy transfer mechanism between UCNPs and GQDs. Reproduced with permission.^[^ ^95^ ^]^ Copyright 2016, American Chemical Society.

Homogeneous assay is a liquid‐phase assay where the hybridization from cognition process between probes and targets to signal readout is completed in the solution, which is a rapid detection method with no need for separation and tedious washing process.^[^ ^88^ ^]^ In this system, the majority of functionalized‐UCNPs usually act as donors to transfer the energy to adjacent modified‐acceptors by the base pairs complementary principle or antigen‐antibody recognition mechanism when adding viral targets.^[^ ^89^, ^90^, ^91^ ^]^ The system's detecting capabilities are demonstrated by the quenching efficiency at varying concentrations of targets. In fact, a variety of parameters, including donor‐acceptor type, the distance between donor‐acceptor pairs, and connection format between targets and donor‐acceptor pairs, might affect detection sensitivity. In earlier years, our team conceived of the idea of researching virus detection using UCNPs and constructed a biosensor based on BaGdF_5_:Yb/Er UCNPs and Au NPs to detect Avian Influenza Virus H7 Subtype with a low LOD of 7 pM (Figure 6A).^[^ ^89^ ^]^ The complementary oligo modified‐UCNPs conjugated with target modified‐Au NPs at a close distance for around 2 h, resulting in the fluorescent quenching of UCNPs. In parallel, Wu et al. developed a UCNP‐based biosensor for HIV antibodies detection in human serum by using GO as the acceptor.^[^ ^92^ ^]^ Long‐chain DNA possesses great selectivity, it plays a vital role in the confirmation of most diseases. To solve the problem of low sensitivity in long‐chain DNA, a ‘head‐to‐tail’ sandwich strategy was proposed by Liu et al. to detect the HIV virus.^[^ ^93^ ^]^ In their strategy, the ‘head‐to‐tail’ format shortens the distance between UCNPs and Au NPs, which facilitates the quenching efficiency, improving the sensitivity compared with the conventional ‘tail‐to‐tail’ system. This technique provides a new platform for long‐chain DNA detection, which is valuable in clinical diagnosis.

To achieve better performance, our group further developed an UCNPs‐target‐Au NPs sandwich assay for detecting the influenza H7 subtype with a low LOD of 134 × 10^−12^ m, featuring high sensitivity and rapidity (Figure 6B).^[^ ^94^ ^]^ This sandwich structure avoids the pre‐treatment of the target with probes, and thus the target could be directly hybridized with probes, reducing the incubation time to 40 min. Besides, the use of an 808 nm light source and the introduction of Nd^3+^ ions in this system protect the DNA from the damage of overheating caused by 980 nm. Normally, most acceptors like QDs and Au NPs are quenchers in UCNP‐based biosensors, this kind of biosensor mainly relies on the quenching efficiency to quantify targets, and thus the sensitivity is relative to the intrinsic luminescent intensity of UCNPs. Interestingly, GQDs have a broad NIR emission and could absorb NIR light sources. Meanwhile, this class of nanomaterials has a relatively high quantum yield. Putting these features together, Marco et al. developed a highly sensitive biosensor based on visible‐to‐NIR fluorescent GQDs as donors and NaYF_4_:Yb, Er@SiO_2_ as acceptors for detecting miRNA sequence (DENV‐2‐vsRNA5) from Dengue illness, facilitating a high sensitivity with a low LOD in the femtomole level (Figure 6C,D).^[^ ^95^ ^]^ In their report, GQDs act as an antenna to transfer these quanta to UCNPs after absorbing 980 nm when they link with UCNPs in close proximity by the π‐π stacking interaction, resulting in a large enhancement in UCL intensity. With the presence of targets, a more stable double‐strand DNA was formed in UCNPs and targets, which hampered the conjugation between GQDs and UCNPs, which could quantify the target according to the reduction of UC luminescent enhancement. This immunoassay greatly facilitates enhancing the sensitivity through improving UC luminescent efficiency, and thus it paves a way for rapid, ultrasensitive, cost‐effective bioassay platform. Therefore, various methods demonstrate that FRET‐based homogenous assays have good potential for virus detection and certainly there is room to improve detection performances.

#### 3.2.2 Heterogeneous assay

**FIGURE 7:**
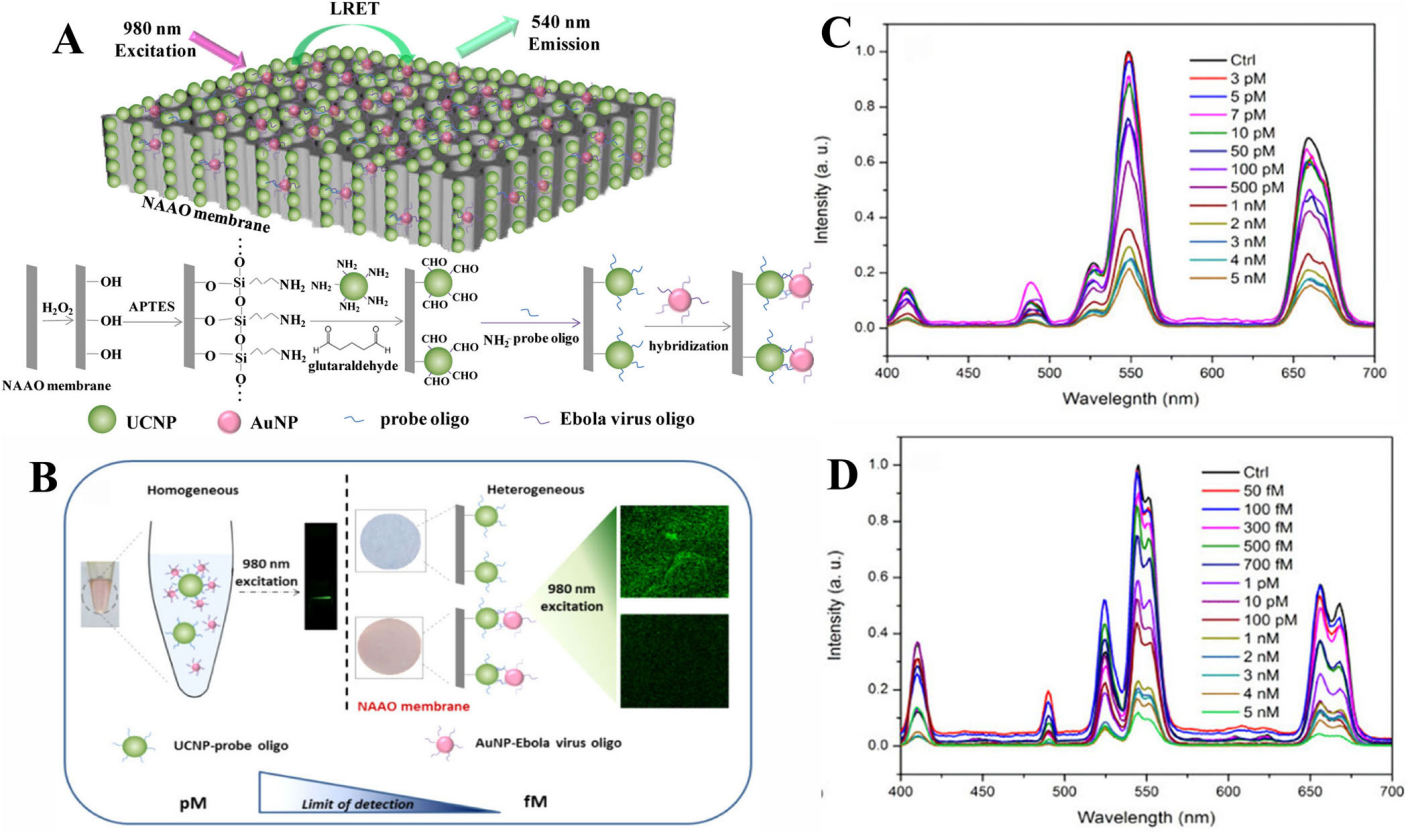
Schematic illustration of heterogeneous upconversion nanoparticle (UCNP)‐based biosensors with FRET effect. Reproduced with permission.^[^ ^97^ ^]^ Copyright 2016, American Chemical Society. (A) The heterogeneous design principle based on UCNPs and Au NPs for Ebola virus oligo detection. (B) Comparison of the homogeneous and heterogeneous assay for Ebola virus detection. (C,D) Emission spectrums of UCNPs with different targets concentration in homogeneous assay and heterogeneous assay with NAAO membrane, respectively.

Heterogeneous assay is a solid‐phase assay where probes and targets are absorbed on the solid substrate with an amount of affinity site, featuring high sensitivity and selectivity. The solid substrates in the heterogeneous assay usually have a high surface‐to‐volume ratio, thus the assay may provide more affinity sites to a large number of probes. Moreover, the surface of the solid substrate is easy to be modified so that it can strongly bind with biomolecules with high specificity.^[^ ^96^ ^]^ Based on these features, UCNP‐based heterogeneous assay under FRET mechanism shows remarkable potential in improving sensitivity for virus detection. To illustrate the advantages of heterogeneous assay in terms of sensitivity, our team previously developed an ultrasensitive biosensor based on BaGdF_5_:Yb/Er UCNPs, Au NPs, and nanoporous membrane system for Ebola virus oligonucleotide detection (Figure 7A–D).^[^ ^97^ ^]^ Nanoporous alumina (NAAO) with three‐dimensional (3D) array structures was introduced in our strategies as a solid substrate which provides more affinity sites throughout the wall of the pillows for capturing UCNPs, resulting in enhancing the sensitivity of UCL. More importantly, we made a comparison for the Ebola virus sensitivity in homogeneous and heterogeneous assays. When adding targets, UCNP‐based heterogeneous assay featured higher quenching efficiency and higher sensitivity with a low LOD of 300 fM. It suggests that the large surface‐to‐volume ratio of this heterogeneous nanoporous membrane assay yielded such a low limit of detection. Besides, this testing can be completed within 45 min without the amplification and separation process. Hence, our technique provides a biosensing platform that could be extended to diagnosing the presence of gene from various viruses with ultrasensitivity, high specificity, and rapidity. Apparently, this solid substrate‐based heterogeneous assay has additional merits of portability and easy operation. Therefore, our result provides a new avenue for early‐stage viral detection based on heterogeneous assay via UC emission. In the heterogeneous assay, it should be noted that the accuracy of detection is dependent on some key parameters, including uniformity, thickness, and density of UCNPs probes on the solid surface. More works are required to optimize these key parameters in the future.

### 3.3 Bioassay through PEC response

**FIGURE 8:**
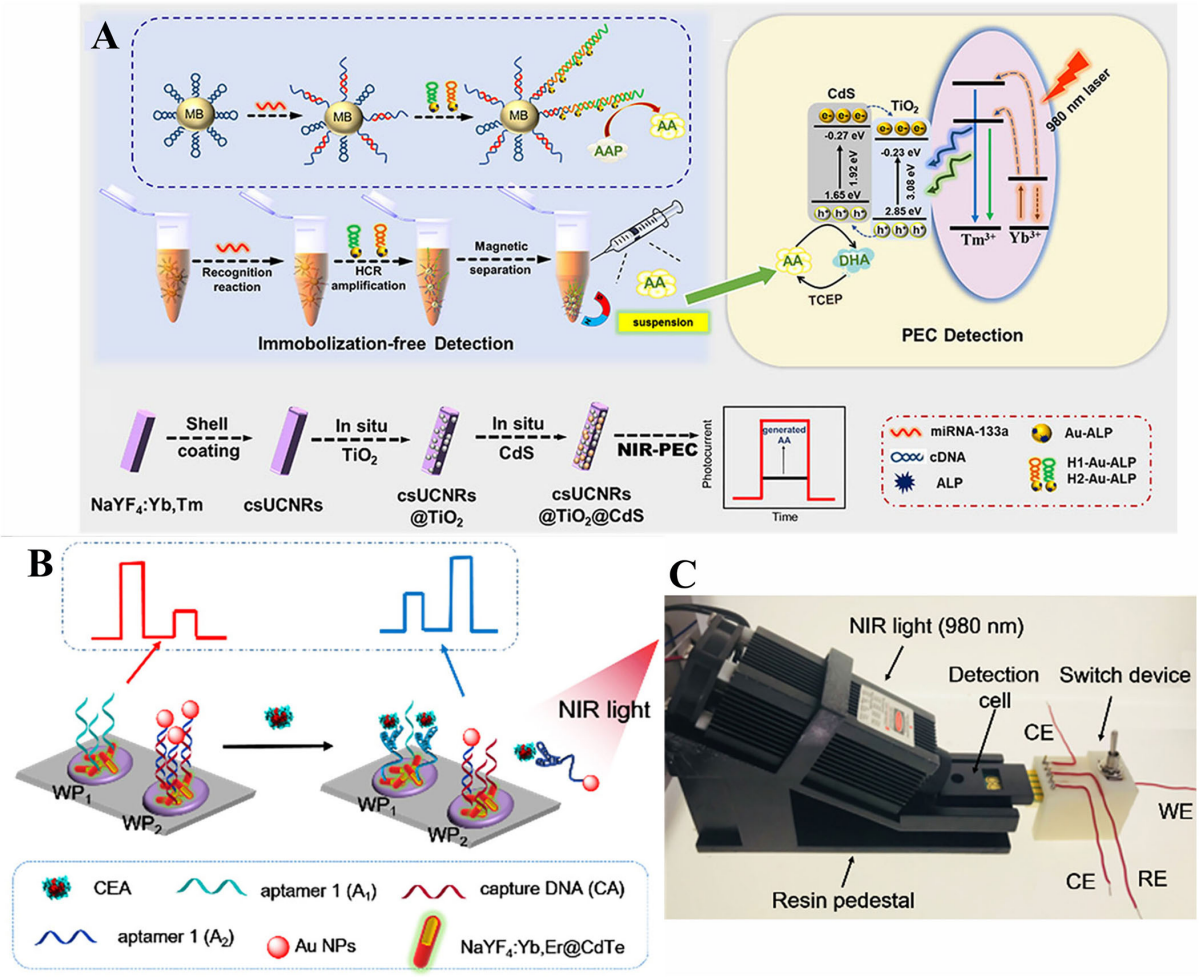
Schematic illustration of upconversion nanoparticle (UCNP)‐based biosensors with PEC effect. (A) The design principle and the energy transfer mechanism of UCNP‐based biosensor with PEC effect for miRNA detection. Reproduced with permission.^[^ ^98^ ^]^ Copyright 2021, American Chemical Society. (B) The design principle of the upconversion‐mediated ratiometric PEC aptasensor for carcinoembryonic antigen detection. (C) Photography of the homemade testing device. Reproduced with permission.^[^ ^99^ ^]^ Copyright 2019, American Chemical Society.

Recently, PEC sensing as an innovative biomolecules analytical tool has sparked widespread attention and it is promising in RNA/DNA and antigen detection due to its excellent characteristics, such as simple equipment needed in the testing, low cost, and low background signal. In this sensing technique, the excitation light source signal could be efficiently segregated from the detection photocurrent signal, preventing the interference of cross signal and leading to a great improvement in sensitivity. The PEC sensing system based on UCNPs with multiple energy states overcomes the wide‐band barriers of photoactive materials and low utilization of UV. Here UCNPs show high optical and chemical stability, which could avoid environmental interference. Based on the above merits, it was reported that NaYF_4_:Yb, Tm@NaYF_4_@TiO_2_@CdS core–shell‐shell structure was constructed to serve as the photoactive materials for miRNA biosensing (Figure 8A).^[^ ^98^ ^]^ TiO_2_ and CdS worked as semiconductors owing to their matching energy levels, which could accelerate electron transfer and suppress charge recombination. To improve the sensitivity of the biosensor, a non‐immobilization method with a dual amplified signal by a hybridization chain reaction and redox circle strategy was introduced, which may minimize the steric hindrance effect from targets on the surface of electrodes. Benefiting from its photocurrent enhancement, this technique showed a sensitive LOD as low as 36.12 aM with high selectivity and reproducibility. To improve the anti‐interference properties, Qiu et al. proposed a ratiometric spatial‐resolution biosensor based on NaYF_4_:Yb, Tm@NaYF_4,_ and CdTe for carcinoembryonic antigen detection (Figure 8B).^[^ ^99^ ^]^ In their approach, two adjacent working electrodes with different bioconjugation were used, aiming at a dual signal readout. Upon adding targets, photocurrent response in WP1 decreased due to the steric hindrance effect while WP2 presented an opposite trend owing to the exciton−plasmon interactions. The ratio of photocurrent intensity of WP1 to WP2 was linear to the target concentration with a low LOD of 4.8 pg/ml under 980 nm excitation, which eliminated the background fluctuation and featured high sensitivity. More importantly, they fabricated a portable and miniaturized testing device by 3D printing technology, as shown in Figure 8C, which provided an accessible and promising way in clinic application. Some researchers have developed various PEC sensing platforms to improve sensitivity for antigen detection.^[^ ^42^, ^58^, ^100^, ^101^ ^]^ For instance, plasmonic gold between UCNPs and CdS was employed to improve electron–hole pairs generation, and then a dual‐readout biosensing platform with PEC and NIR‐induced fluorescent visualization format was introduced to improve the detection speed with high veracity of analysis. Therefore, those PEC‐based biosensors feature high sensitivity and selectivity in detecting nucleic acid, antigen, and antibody. However, there are some factors limiting the sensing capability of these PEC platforms. For instance, there is less effort in improving the assembling technique of photoactive materials on the electrode, which usually has a low assemble efficiency and a high risk of falling off from the electrode. Therefore, PEC sensors usually have low stability and reproducibility, preventing them from being used in practical applications. Moreover, the detection accuracy may be influenced by the position and distance between electrodes.

### 3.4 SPR‐based bioassay

**FIGURE 9:**
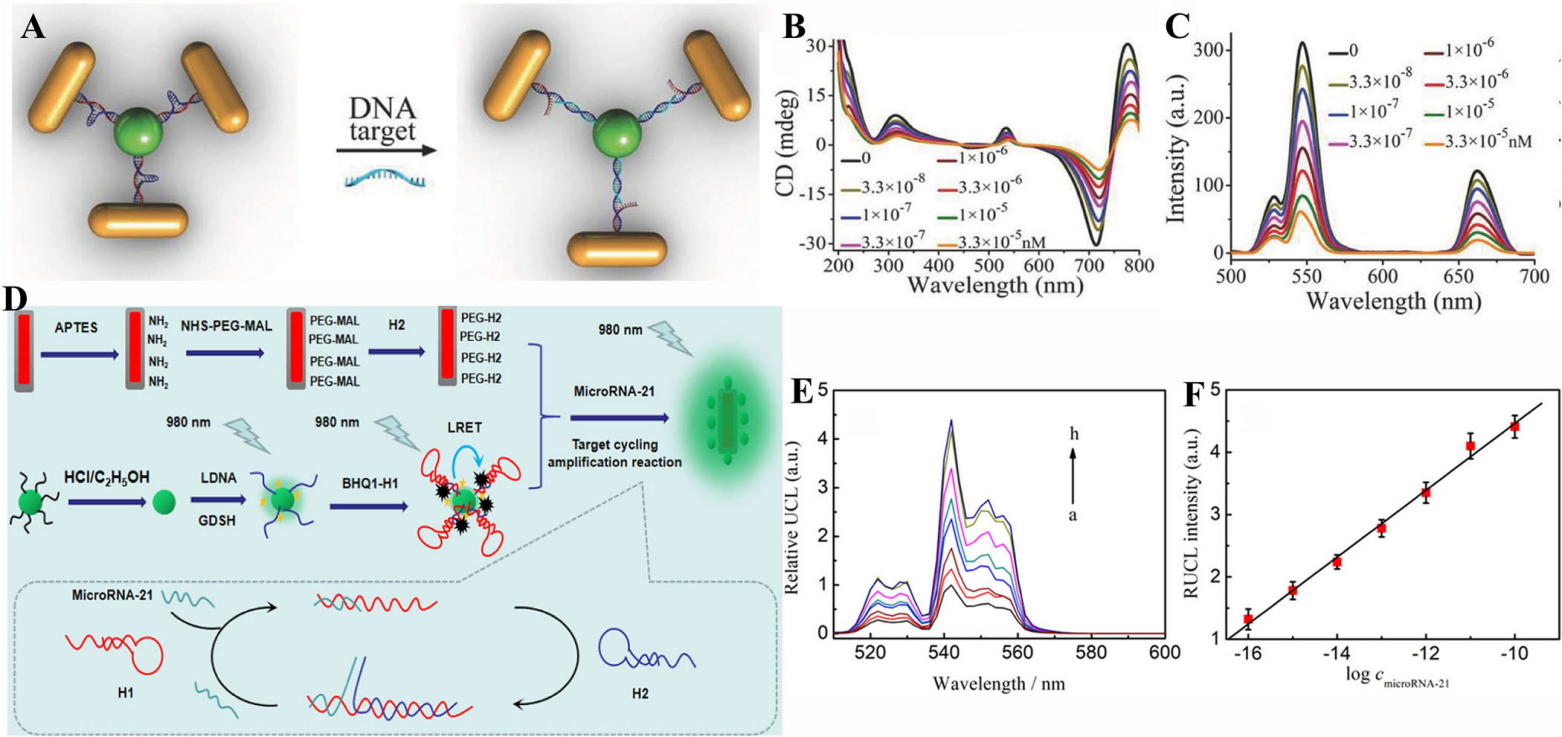
Schematic illustration of upconversion nanoparticle (UCNP)‐based biosensors with SPR effect. (A) Schematic illustration of DNA biosensing mechanism based on UCNPs‐Au nanorods tetramer. (B,C) The circular dichroism and upconversion luminescence (UCL) spectrum with different concentrations of DNA. (D) The design principle of biosensors based on UCNPs and Au nanorods. Reproduced with permission.^[^ ^102^ ^]^ Copyright 2016, Wiley Online Library. (E) The UC emission spectra with different target concentrations. (F) The line relationship between UCL intensity and the logarithm of microRNA‐21 concentration. Reproduced with permission.^[^ ^103^ ^]^ Copyright 2020, American Chemical Society.

SPR‐based bioassay is a conventional analytical technology based on the noble metal nanostructure. Many studies demonstrated that SPR‐based biosensors can be applied in virus detection with high sensitivity, and most biosensors operate based on the surface‐enhanced Raman scattering (SERS) effect. Interestingly, coupling UCNPs with metal nanomaterials can largely improve UCL intensity, which is beneficial for boosting sensitivity of UCNP‐based biosensors. Moreover, UCNPs and metal nanostructure are readily accommodated and further modified with nucleic acid, aptamers, antibodies, and antigens. Based on these features, UCNP‐based biosensor coupled with surface plasmon is a potential candidate for virus and biomolecule detection. Interestingly, Wu and co‐workers fabricated a propeller‐like tetramer assembled by Au nanorods and UCNPs for hepatitis A virus Vall7 polyprotein gene (HVA) detection, as shown in Figure 9A–C.^[^ ^102^ ^]^ In their work, one innovation is the fabrication of the propeller‐like tetramer, vastly enhancing the UCL intensity up to 21.3‐folds, which was ascribed to the plasmon resonance enhancement to UCL. Based on the propeller‐like structure, two partially complementary DNA sequence‐modified UCNPs and Au nanorods were conjugated in a hairpin‐like format. Upon the presence of oligonucleotide targets, the hairpin‐like structures were extended because of the specific recognition. Thus, the gap between them became longer, resulting in a change in signals. Besides, they used UCL and circular dichroism (CD) from metal nanomaterials‐mediated assemblies as the dual‐signal to detect the HVA, which featured high sensitivity with a low LOD of 20.3 and 13.2 aM, respectively. Recently, Zhang et al. developed an ultrasensitive sensor based on NaYF_4_@NaYF_4_:Yb, Er@NaYF_4,_ and Au nanorods for microRNA‐21 detection (Figure 9D–F).^[^ ^103^ ^]^ The SiO_2_ shell on the Au nanorod's surface acts as an insulation to avoid the luminescent quenching from the energy transfer or non‐radiation decay, which could guarantee a high efficiency of plasmonic interaction between Au nanorods and UCNPs. Furthermore, this system adopted a measure of target cycling amplification reaction with surface plasmon. Briefly, the luminescent quenching from the coating of BHQ1‐labeled hairpin DNA (BHQ1‐H1) on UCNPs was recovered and then luminescence was further enhanced due to the plasmon resonance effect after adding the analysts. This microRNA‐based sensing presented a high sensitivity with a low LOD of 0.036 fM, and a linear range from the 0.1 fM–0.1 nM with high selectivity and stability. Furthermore, this sensor was applied in microRNA‐21 expression cells and clinical serum samples, implying a high potential in virus detection. However, there are still some challenges in the surface plasmon‐based biosensors combining with UCNPs. For example, it is difficult to precisely manipulate the distance between UCNPs and metal plasmon, and the shape and size of metal plasmon are the influence factors that should be carefully considered.

## 4 CONCLUSION AND OUTLOOK

In summary, we provide an overview of recent development of UCNP‐based biosensors for virus detection, which enjoy high sensitivity, rapidity and reliability benefited from UCNPs' unique characteristics of low autofluorescence and minimized background signal. This Review summarized the design principle of UCNP‐based biosensors in four major categories: 1) intrinsic luminescence changes of UCNPs, 2) luminescence with FRET effect, 3) luminescence with SPR effect, and 4) luminescence with electrochemistry. These strategies are currently capable of demonstrating the enhancement in UCNP‐based biosensors’ performance for viral detection. Some of the LOD could be as low as aM (10^−18^ m) level, which is significantly more sensitive compared to many conventional detection methods. More importantly, considering the figures of merit to assess the capability of biosensors (e.g., sensitivity, specificity), UCNP‐based biosensors demonstrate excellent sensor performance with additional benefits of low cost, convenience, and high throughput compared with conventional methods such as Au NPs in LFIAs and fluorescent dyes in microarrays. We also summarized different methodologies for detecting nucleic acid, antibody, and antigen utilizing UCNP‐based biosensors.

Future works should focus on exploring new UCNP‐based biosensors, such as introducing innovative functional nanomaterials and detection principles. Developing new strategies to improve sensing capabilities is imperative, making UCNPs an alternative and reliable nanophosphor suitable for virus detection technique. To date, there are some key challenging issues in this research field. First, the efficiency and the linearity of the response from UCNPs can be further improved to provide more concise and accurate detection results. Furthermore, in the context of FRET, the distance between donor‐acceptor pairs and the size of UCNPs have a major impact on the reliability of the detection readout, whereas the size of metal plasmon in SPR‐based detection would pose a significant effect on the detection capability. Thereby, a more precise manipulation of these factors during the synthesis process of UCNP and metallic NP will enhance the performance of these biosensors. The complication in long‐chain DNA detection has hindered the development and application of such a method. The integrated UCNP‐based biosensor may resolve relevant problems and result in more sensitive and reliable DNA detection. Last, the rational processes of UCNP synthetization and surface modification are vital in biosensing applications. These complications and time‐consuming processes are in urgent need of optimization.

## CONFLICT OF INTEREST

The authors declare no conflict of interest.
